# Seasonal analysis of match load in professional soccer players: An observational cohort study of a Swiss U18, U21 and first team

**DOI:** 10.3389/fphys.2022.1023378

**Published:** 2023-01-04

**Authors:** Linda Ammann, Stefan Altmann, Ludwig Ruf, Billy Sperlich

**Affiliations:** ^1^ Integrative and Experimental Exercise Science, Department of Sport Science, University of Würzburg, Würzburg, Germany; ^2^ Institute of Sports and Sports Science, Karlsruhe Institute of Technology, Karlsruhe, Germany; ^3^ TSG ResearchLab gGmbH, Zuzenhausen, Germany

**Keywords:** load management, soccer, match, physical performance, long-term development, maturation, team sports

## Abstract

The aim of this study was to quantify and compare various external match load measures in three age groups and leagues in male soccer (U18 in highest league of their age group vs U21 in fourth highest league vs first team in highest league). In this retrospective observational cohort study accelerations, decelerations, absolute and relative high-speed running as well as sprint distance, dynamic stress load, explosive distance, high intensity bursts total distance, high metabolic load (HML) distance, speed intensity, total distance, total time, and total loading were assessed in 416 individual player matches of 59 players. All these external load measures showed large inter-individual variability. At a group level, one-way ANOVAs or Kruskal–Wallis tests revealed statistically significant differences between the three teams for all measures analyzed (all *p* < 0.05), except accelerations. The first team displayed statistically significant higher dynamic stress load, explosive distance, HML distance, speed intensity, total distance and total loading compared to the two youth teams (all *p* < 0.05). The U18 featured statistically significant higher number of decelerations, absolute and relative high-speed running distance, high metabolic load distance, speed intensity, relative sprint distance, total distance, and total time than the U21, while for U21 higher dynamic stress load was observed than for U18 (all *p* < 0.05). Based on our data we conclude a routinely monitoring of match loads of different age groups and competitive settings to be required to 1) provide an indication of what players need to be prepared for, 2) track the athletic and match evolution, and 3) individually tailor training programs allowing players to fulfill the short- and long-term sport-specific requirements.

## Introduction

As physical performance is an important component of soccer match performance (Julian, Page, & Harper, 2020; Forcher et al., 2022b; Dambroz, Clemente, & Teoldo, 2022; Sarmento et al., 2022), the aim is to optimize these capabilities. In order to individually tailor training programs maximizing positive physiological adaptation and at the same time preventing injury and illness, careful load monitoring is required (Halson, 2014; Akenhead & Nassis, 2016; Bourdon et al., 2017; Impellizzeri et al., 2019; Miguel et al., 2021). The various measures to judge training or match load are categorized as either internal (e.g., heart rate-based measures, session rating of perceived exertion) or external (e.g., distance related measures, impacts, accelerations), depending on whether they refer to measurable aspects occurring internally or externally to the player (Bourdon et al., 2017; Impellizzeri et al., 2019; Miguel, Oliveira, Loureiro, Gracia-Rubio, & Ibanez, 2021).

Advancements in technology made it possible to more accurately measure internal and external loads and thus the physical demands of soccer training and matches can now be better quantified (Halson, 2014; Bourdon et al., 2017; Palucci Vieira, Carling, Barbieri, Aquino, & Santiago, 2019; Miguel et al., 2021; Torres-Ronda et al., 2022). Numerous studies assessed match loads of elite male, female, and youth soccer players regarding physical performance capacities, playing positions, tactical formation, or different contextual factors (e.g., match location, environmental conditions, match importance, preparation, fixture congestion, season phase, match outcome, nutrition strategies, game rules) (Link & de Lorenzo, 2016; Palucci Vieira et al., 2019; Julian, Page, & Harper, 2020; Altmann et al., 2021; Chmura et al., 2021; Forcher et al., 2022a; Forcher et al., 2022b; Draper et al., 2022; Harkness-Armstrong, Till, Datson, Myhill, & Emmonds, 2022; Hulton, Malone, Clarke, & MacLaren, 2022; Sarmento et al., 2022). While the findings, especially at elite level, are not consistent, it is evident that data from one population group may not be relevant to other population groups (Palucci Vieira et al., 2019; Saeterbakken et al., 2019; Reynolds et al., 2021; Forcher et al., 2022a). At the youth level, the available data from few investigations indicate an increase of physical match loads with age, for both male and female soccer players (Palucci Vieira et al., 2019; Reynolds et al., 2021; Harkness-Armstrong et al., 2022). Furthermore, previous research provided evidence that matches, at least for regular players, are the most demanding sessions within a microcycle (Stevens, de Ruiter, Twisk, Savelsbergh, & Beek, 2017; Oliva-Lozano, Gomez-Carmona, Fortes, & Pino-Ortega, 2022) as well as the load, especially related to intense running, in elite soccer matches has increased in recent years (Barnes, Archer, Hogg, Bush, & Bradley, 2014; Bush, Barnes, Archer, Hogg, & Bradley, 2015; Chmura et al., 2018; Chmura et al., 2019; Altmann et al., 2021; Pons et al., 2021).

In order to identify and develop the physiological aspects relevant to soccer performance, it seems essential for coaches, supporting staff and researchers in the field to have a solid understanding about the current (internal and external) match load characteristics for players at different ages and in different competitive settings, requiring constant re-evaluation. To the best of our knowledge, the match loads in Swiss men’s soccer, both at elite and performance-oriented youth level, have not yet been examined. Thus, the aim of this study was to quantify and compare typical external load match demands of Swiss male soccer in three teams with different age group respective league (U18 in highest league of their age group vs U21 in fourth highest league vs first team in highest league) using global navigation satellite system (GNSS) technology.

## Materials and methods

### Participants

A total of *N* = 59 male professional soccer field players from a Swiss club were asked to take part in this study. The goalkeepers were excluded due to their different activity profile compared to field positions (White et al., 2018). The eligible players were screened for health contraindications by the internal club sports medicine staff as part of their usual care of the players, which meant that the only criterion for inclusion was to have been fielded over full duration of at least one match considered during the data collection period. The players were asked whether data measured before the researchers had the opportunity to ask them to participate could also be included in the analysis. A total of three additional players were also fielded for the entire duration of at least one match during the mentioned period, but since they had left the club in the meantime, they were not asked to participate, and their data (potential individual match observations) were excluded from analysis (*n* = 9). All of the requested participants and their guardians voluntarily gave their written informed consent. As all data used in the current study arose from routine monitoring, no ethical approval was required (Winter & Maughan, 2009). A descriptive characterization of the participants is summarized in Table 1.

**TABLE 1:** Descriptive statistics of the participant’s age, body height, body mass and maximal speed. Data presented as mean (SD) and range.

External load measure [unit] and statistical indicator	U18 (*n* = 25)	U21 (*n* = 24)	1st team (*n* = 19)
Age [years]			
Mean (SD)	17.41 (0.67)	19.50 (2.03)	26.20 (4.38)
Range	15.8 to 18.8	17.2 to 24.7	19.5 to 36.1
Body height [m]			
Mean (SD)	1.784 (0.051)	1.820 (0.048)	1.818 (0.054)
Range	1.68 to 1.90	1.66 to 1.90	1.74 to 1.95
Body mass [kg]			
Mean (SD)	70.72 (6.23)	76.39 (6.00)	77.17 (7.39)
Range	60.4 to 83.0	64.0 to 86.0	66.0 to 89.0
Maximal speed [km/h]			
Mean (SD)	33.400 (1.054)	33.577 (0.997)	33.384 (1.312)
Range	31.25 to 35.78	31.90 to 35.60	31.21 to 36.32

### Study design and research methods

A retrospective observational cohort study was implemented on three male teams (under 18 years (U18), under 21 years (U21) and first team) of a professional soccer club in Switzerland over a 39 week-period during the 2021–2022 season. This period, lasting until the end of the season, was chosen because consistent measurement methods were available at that time. In the observational period the first team competed in the highest national championship (Credit Suisse Super League) as well as the national cup competition (Helvetia Schweizer Cup). In addition to 32 championship matches and four cup matches, there were also six test matches scheduled. The U21 team played 23 matches in the championship “1. Liga” (i.e., the fourth highest and not age-restricted league in Switzerland), and six test matches. The U18 team played 26 championship and four cup matches, with both competitions being the highest organized for their age group. In addition, there were five test matches for this team.

The data analyzed were recorded as part of the daily routine monitoring of the players and analyzed *a posteriori*. This study did not influence or alter the sessions in any way. Only matches of the official championships were analyzed (*n* = 81). They were played on either natural or artificial turf pitches and the match format was always two-halves of 45 min, separated by a 15-min break. Of these matches, those were excluded from the analysis in which there were less than 10 field players in the investigated team at the final whistle (*n* = 7). In addition, only players having played for the entire duration of the match were included in the analysis, resulting in 423 individual player match observations. In these, only match time was considered (i.e., all activities before kick-off, during half-time and after the end of the match were excluded). The data of an individual player match observation were excluded when incomplete (*n* = 7). Such missing values could be due to technical or practical problems with the GNSS devices. The players were divided into the teams U18 (*n* = 25), U21 (*n* = 24), and first team (*n* = 19) depending on the team they played for in the respective match. The fact that some players were fielded in more than one team explains why the sum of the players in the three teams is greater than the above-mentioned number of participants. In addition, as allowed by the regulations, at earlier stages of the season, some players who had already reached the age of 21 played in the U21 team. The final analysis comprised of 74 matches (U18 = 22, U21 = 23, and first team = 29) and 416 individual player match observations (U18 = 121, U21 = 133, and first team = 162; by player: *M* = 7.1, *SD* = 6.3, range 1 to 27). Their distribution among positions and match outcomes are summarized in Tables 3–6. Five different playing positions were categorized (central defender, full back, central midfielder, wide midfielder, forward) (Chmura et al., 2021; Forcher et al., 2022a; Modric et al., 2022). For the first team, the definitions were made in consultation with an assistant coach directly involved in the line-up and substitutions. In case of the youth teams, the written reports of the head coach or athletic coaches present were employed. It is worth noting that the first team often played in a 4-4-2 diamond tactical formation and, due to the intended position interpretation of the players, we deemed appropriate to define all four midfielders as central midfielder. For this analysis, the positions of the players at the start of the match were used. 18 situations were reported in which a player had to adopt a different position during the course of a match.

### External load

A variety of external load measures were monitored for each player using global navigation satellite system (GNSS) technology (Apex Pro, STATSports, Newry, Ireland) with 10 Hz sampling rate. The validity and reliability of the STATSports Apex 10 Hz system was previously reported elsewhere (Beato, Coratella, Stiff, & Iacono, 2018; Beato & de Keijzer, 2019; Crang et al., 2022). Apex10 Hz is a multi GNSS augmented unit, capable of acquiring and tracking multiple satellite systems (e.g., GPS, GLONASS, Galileo, BeiDou) concurrently to provide the best possible position information. The Apex GNSS model reports information about the number of satellites the GNSS receiver is interacting with while calculating the position of the GNSS unit (*M* = 18.1, *SD* = 1.4, range 14 to 21), which was slightly lower than reported in previous literature (Beato et al., 2018; Beato & de Keijzer, 2019; Reynolds, Connor, Jamil, & Beato, 2021). The Apex units used present the following characteristics: dimension 30 mm (wide) × 80 mm (high), weight 48 g, 100 Hz gyroscope, 100 Hz tri-axial accelerometer, and 10 Hz magnetometer. For each player an Apex unit was placed, according to manufacturer’s instructions, on the upper back between the right and left scapula through a vest. After data collection on the pitch, the Sonra software (Sonra 4.0, STATSports, Newry, Ireland) was used to download all data recorded by the GNSS and precisely define each player’s playing time. The data was then exported as a csv file for further analysis. To avoid inter-unit errors, players wore the same GNSS device and vest in each session (Bourdon et al., 2017).

The 14 external load measures listed in Table 2 were selected for analysis. All distance-related measures, accelerations, and decelerations, all of them with their respective thresholds, were selected because they have been used most frequently in practice and in studies analyzing external load (especially in soccer), and literature proposes to consider them (Rago, Brito, Figueiredo, Krustrup, & Rebelo, 2019; Rago, Brito, Figueiredo, Krustrup, & Rebelo, 2020; Miguel et al., 2021; Teixeira et al., 2021). The latter also applies to total loading and total time. In line with other studies (Miguel et al., 2021; Reynolds et al., 2021), also high metabolic load (HML) distance [m], explosive distance [m], high intensity bursts (HIB) total distance [m], speed intensity and dynamic stress load were assessed. The percentage thresholds for the relative speed thresholds (i.e., 55 and 70) are explained by the fact that they correspond to the recommended fixed thresholds (Miguel et al., 2021) for a maximum speed of 36 km/h. In the present analysis, the individual maximum speed was defined as the respective highest speed measured by GNSS (Massard, Egger, & Lovell, 2019) during training or match, provided it followed a proper acceleration phase, the absence of which reveals clear measurement errors. In case a new maximum speed was measured, the new value replaced the previous one. In the training sessions of the two youth teams, training exercises aiming at reaching the individual maximum speed were integrated at regular frequencies.

**TABLE 2:** Definitions and units of the external load measures included in the analysis. a.u. = arbitrary units.

External load measure	Unit	Definition
Accelerations	[n]	Acceleration efforts performed between 4 and 10 m/s^2^ with a minimum duration of 0.5 s.
Decelerations	[n]	Deceleration efforts performed between 4 and 10 m/s^2^ with a minimum duration of 0.5 s.
Dynamic stress load	[a.u.]	The total of the weighted impacts, which is based on accelerometer values of magnitude above 2 g. This measure weights the impacts using a convex-shaped function. The aggregated weighted impacts are scaled to provide more workable values.
Explosive distance	[m]	The distance [m] covered by a player with a metabolic power >25.5 W/kg, but with a velocity <5.5 m/s.
High intensity bursts (HIB) total distance	[m]	A high intensity burst is defined as any time whereby a minimum of three high-intensity activities (acceleration ≥3.5 m/s^2^, deceleration ≥3.5 m/s^2^, or impacts ≥11 g) occurred separated by 20 s or less. High intensity burst total distance refers to the number of meters covered under these conditions.
High metabolic load distance	[m]	The distance [m] covered by a player performing any activity with a metabolic power (energy consumption per kilogram per second) ≥ 25.5 W/kg for at least 1 s.
High-speed distance		
absolute	[m]	Distance ≥19.8 km/h (5.5 m/s).
relative	[m]	Distance ≥55% of individual maximal speed.
Speed intensity	[a.u.]	A measure of total exertion calculated as the sum of a convexly weighted measure of instantaneous speed.
Sprint distance		
absolute	[m]	Distance ≥25.2 km/h (7 m/s).
relative	[m]	Distance ≥70% of individual maximal speed.
Total distance	[m]	Total distance covered.
Total loading	[a.u.]	The total of the forces on the player over the entire session based on accelerometer data alone and without any weightings. It uses the magnitude of the accelerometer values taken in three directions, sampled with 100 Hz. The total is scaled by 1000 to provide more workable values.
Total time	[min]	Total playing time.

### Statistical analyses

All data were analyzed with the open-source software RStudio (R version 4.2.0 (2022–04-22 ucrt), R Core Team 2021, Wien, Austria). Descriptive statistics were used to describe and characterize the sample. Thereby mean (SD) and range was reported. Individual player match observations were analyzed without any weighting (e.g., no aggregation per match or player). Descriptive statistics are presented for all external load measures included in the analysis. The distribution, the median, the first and third quartiles (the 25th and 75th percentiles) of each of these measures were presented visually per team; in addition, mean (SD) was reported.

Normal distribution of the dependent variables was assessed employing Q-Q-plots. Levene-test and F-max-test were used to check the homogeneity of variances of the dependent variables. For each dependent variable assumed to be normally distributed, first, a univariate one-way ANOVA without repeated measures test was conducted to evaluate between group differences. Type 3 of sums of squares was used. The effect size generalized eta squared was computed. Multiple pairwise two-sided t-tests with Bonferroni corrections for multiple testing were then calculated for each of these variables. Effect size Cohen’s *d* with 95% confidence interval was determined for each comparison using a pooled SD (Cohen, 1988). For each dependent variable for which no normal distribution could be assumed, a Kruskal–Wallis rank sum test followed by a two-sided Wilcoxon rank sum test with Bonferroni corrections for multiple testing were computed to evaluate between group differences. Eta squared based on the H-statistic with 95% CI was calculated as effect size for Kruskal–Wallis rank sum tests (Tomczak & Tomczak, 2014). The correlation coefficient *r* was computed as effect size for the Wilcoxon rank sum tests as Z statistic divided by square root of the sample size (Tomczak & Tomczak, 2014). The 95% CI for Cohen’s *d*, eta squared of Kruskal–Wallis test and *r* were estimated using a bootstrap method (bootstrap percentile method with 1′000 random bootstrap samples). The level of significance was set at *p* < 0.05 for all tests.

## Results

Selected descriptive statistics of the participants grouped by team assignment are summarized in Table 1. The reported data refer to the first appearance of a player with the team. Table 3 provides the number of the individual player match observations among match outcome grouped for each team. Their distribution over the number of unique matches is summarized in Table 4.

**TABLE 3:** Number of individual match observations per team and match outcome. Data presented as n (%).

Team	Defeat	Draw	Win
U18 (*n* = 121)	50 (41%)	22 (18%)	49 (40%)
U21 (*n* = 133)	54 (41%)	18 (14%)	61 (46%)
1^st^ (*n* = 162)	46 (28%)	59 (36%)	57 (35%)

**TABLE 4:** Number of unique matches per team and match outcome. Data presented as n (%).

Team	Defeat	Draw	Win
U18 (*n* = 22)	9 (41%)	4 (18%)	9 (41%)
U21 (*n* = 23)	9 (39%)	3 (13%)	11 (48%)
1^st^ (*n* = 29)	9 (31%)	10 (34%)	10 (34%)

Table 5 and Table 6 provide the same information among positions.

**TABLE 5:** Number of individual match observations per team and position. Data presented as n (%).

Team	Central defender	Full back	Central midfielder	Wide midfielder	Forward
U18 (*n* = 121)	28 (23%)	21 (17%)	39 (32%)	20 (17%)	13 (11%)
U21 (*n* = 133)	40 (30%)	35 (26%)	42 (32%)	7 (5%)	9 (7%)
1st (*n* = 162)	50 (31%)	43 (27%)	48 (30%)	7 (4%)	14 (9%)

**TABLE 6:** Number of unique matches per team and position. Data presented as n (%).

Team	Central defender	Full back	Central midfielder	Wide midfielder	Forward
U18 (*n* = 22)	19 (86%)	15 (68%)	22 (100%)	18 (82%)	12 (55%)
U21 (*n* = 23)	23 (100%)	23 (100%)	23 (100%)	7 (30%)	9 (39%)
1st (*n* = 29)	29 (100%)	29 (100%)	28 (97%)	7 (24%)	13 (45%)

**FIGURE 1:**
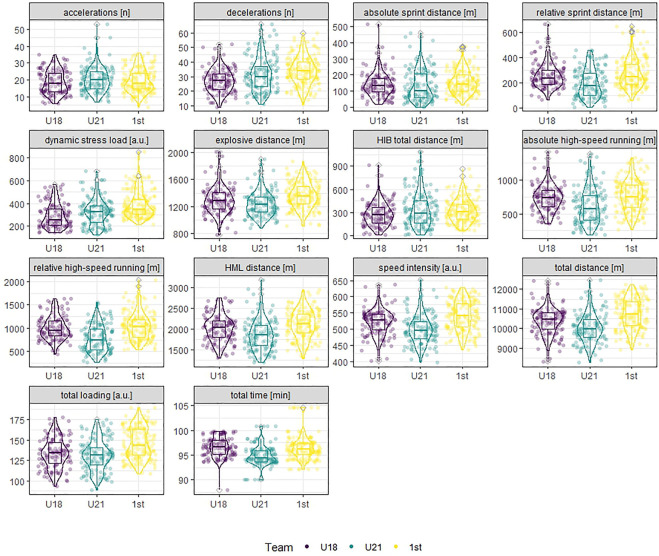
Distribution, median, first and third quartiles (the 25th and 75th percentiles) of each of the external load measures visualized per team. Outliers (values further than 1.5 * inter-quartile range from the hinge) are indicated by grey opened diamonds. a.u. = arbitrary units.

The visualization of the distribution, the median, the first and third quartiles (the 25th and 75th percentiles) of each external load measure among the teams in Figure 1 indicates large inter-individual variability.

The mean and standard deviation from all external load measures analyzed, according to the team clusters are presented in Table 7.

**TABLE 7:** Summary of the U18, U21, and first teams’ match loads. Data presented as mean (SD).

Variable	U18 (*n* = 121)	U21 (*n* = 133)	1^st^ team (*n* = 162)
Total time [min]	96.61 (2.01)	94.92 (2.05)	96.53 (2.52)
Total distance [m]	10,381.5 (678.9)	10,023.7 (740.0)	10,735.7 (765.1)
High-speed running [m]			
absolute	730.6 (194.5)	617.1 (263.2)	761.2 (221.7)
relative	999.5 (247.3)	779.9 (284.5)	1,034.4 (284.8)
Sprint distance [m]			
absolute	141.6 (75.8)	132.7 (96.6)	156.2 (72.9)
relative	257.2 (97.7)	195.5 (112.6)	274.6 (116.4)
Accelerations [n]	18.5 (6.8)	20.3 (7.2)	19.4 (6.7)
Decelerations [n]	27.3 (8.1)	31.1 (11.6)	33.7 (9.5)
Total loading [arb. Units]	134.087 (18.136)	131.101 (18.322)	146.027 (18.984)
HML distance [m]	2,018.2 (313.1)	1,858.3 (368.6)	2,123.6 (338.1)
Explosive distance [m]	1,287.7 (198.4)	1,241.2 (185.9)	1,362.4 (188.3)
HIB total distance [m]	274.2 (150.0)	327.3 (215.4)	320.9 (148.6)
Speed intensity [arb. Units]	521.397 (38.970)	499.815 (44.410)	542.007 (45.258)
Dynamic stress load [arb. Units]	289.211 (105.171)	322.767 (113.703)	384.339 (112.253)

Univariate one-way analysis of variance without repeated measures and Kruskal–Wallis tests revealed statistically significant differences between the three teams for all external load measures (all *p* < 0.05), except for accelerations above 4 m/s^2^. Table 8 and Table 9 provide more detailed information on the magnitude of the differences between the teams. While Table 8 presents the results of the multiple pairwise t-tests with Bonferroni corrections for multiple testing and effect size Cohen’s *d*, Table 9 shows the results of the Wilcoxon rank sum test with Bonferroni corrections for multiple testing and effect size *r*.

**TABLE 8:** T-tests (with Bonferroni corrections) comparisons between teams for selected external load measures. Confidence level of the d confidence intervals = 0.95. Level of significance: *p < 0.05.

Variable	Team comparison		*t*	*df*	*p*	d [95% CI]
Decelerations	1^st^	U18	6.09	275.91	<0.001*	0.72 [0.48 to 0.96]
	1^st^	U21	2.06	254.19	0.121	0.25 [0.00 to 0.48]
	U18	U21	-3.05	236.83	0.008*	-0.38 [-0.62 to -0.14]
High-speed running						
absolute	1^st^	U18	1.23	273.76	0.654	0.15 [-0.10 to 0.40]
	1^st^	U21	5.02	258.63	<0.001*	0.60 [0.35 to 0.85]
	U18	U21	3.93	242.09	<0.001*	0.49 [0.23 to 0.74]
relative	1^st^	U18	1.10	274.64	0.816	0.13 [-0.11 to 0.37]
	1^st^	U21	7.64	282.03	<0.001*	0.89 [0.66 to 1.15]
	U18	U21	6.58	251.49	<0.001*	0.82 [0.58 to 1.09]
HML distance	1^st^	U18	2.71	268.33	0.022*	0.32 [0.11 to 0.57]
	1^st^	U21	6.38	271.21	<0.001*	0.75 [0.52 to 1.03]
	U18	U21	3.74	250.84	<0.001*	0.47 [0.21 to 0.75]
Speed intensity	1^st^	U18	4.11	275.31	<0.001*	0.48 [0.25 to 0.73]
	1^st^	U21	8.05	283.86	<0.001*	0.94 [0.71 to 1.21]
	U18	U21	4.12	251.68	<0.001*	0.51 [0.25 to 0.81]
Total distance	1^st^	U18	4.11	272.73	<0.001*	0.49 [0.25 to 0.76]
	1^st^	U21	8.10	285.23	<0.001*	0.94 [0.72 to 1.21]
	U18	U21	4.02	251.98	<0.001*	0.50 [0.24 to 0.77]
Total loading	1^st^	U18	5.37	264.66	<0.001*	0.64 [0.42 to 0.89]
	1^st^	U21	6.85	285.43	<0.001*	0.80 [0.55 to 1.03]
	U18	U21	1.30	250.20	0.579	0.16 [-0.07 to 0.39]

**TABLE 9:** Wilcoxon rank sum tests (with Bonferroni corrections) comparisons between teams for selected external load measures. Confidence level of the r confidence intervals = 0.95. Level of significance: *p < 0.05.

Variable	Team comparison		*W*	p	r [95% CI]
Accelerations	1^st^	U18	10517.0	0.879	0.06 [0.00 to 0.19]
	1^st^	U21	9978.0	0.825	0.06 [0.00 to 0.18]
	U18	U21	6951.0	0.182	0.12 [0.01 to 0.25]
Sprint distance					
absolute	1^st^	U18	11003.0	0.233	0.10 [0.01 to 0.21]
	1^st^	U21	13274.0	0.002*	0.20 [0.08 to 0.31]
	U18	U21	9067.0	0.243	0.11 [0.01 to 0.24]
relative	1^st^	U18	10444.5	1.000	0.06 [0.00 to 0.18]
	1^st^	U21	14950.0	<0.001*	0.33 [0.22 to 0.43]
	U18	U21	10825.5	<0.001*	0.30 [0.18 to 0.41]
Dynamic stress load	1^st^	U18	14898.5	<0.001*	0.42 [0.32 to 0.52]
	1^st^	U21	13678.0	<0.001*	0.23 [0.12 to 0.34]
	U18	U21	6612.0	0.043*	0.15 [0.03 to 0.28]
Explosive distance	1^st^	U18	12103.5	0.002*	0.20 [0.08 to 0.30]
	1^st^	U21	14898.5	<0.001*	0.33 [0.22 to 0.43]
	U18	U21	9404.5	0.061	0.15 [0.02 to 0.26]
HIB total distance	1^st^	U18	11643.0	0.021*	0.16 [0.05 to 0.27]
	1^st^	U21	11227.0	1.000	0.04 [0.00 to 0.16]
	U18	U21	7178.0	0.414	0.09 [0.01 to 0.22]
Total time	1^st^	U18	8987.0	0.696	0.07 [0.00 to 0.19]
	1^st^	U21	15234.5	<0.001*	0.36 [0.25 to 0.46]
	U18	U21	12073.0	<0.001*	0.43 [0.32 to 0.53]

## Discussion

The aim of this study was to quantify and compare various measures of external match load in three teams with different age group respective league of Swiss male soccer using GNSS technology. The main findings indicated substantial inter-individual variability in all external load measures and all teams differed in all external load measures analyzed (all *p* < 0.05), except for accelerations. Specifically, the first team showed i) higher match loads compared with the U21 players in all external load measures analyzed, except for accelerations, decelerations and HIB total distance; and ii) compared to the U18 players higher number of decelerations, dynamic stress load, HIB total distance, HML distance, explosive distance, speed intensity, total distance and total loading. iii) Interestingly, higher match loads have been observed in the U18 compared to the U21 for decelerations, absolute and relative high-speed running, HML distance, speed intensity, total distance, relative sprint distance and total time; iv) whereas dynamic stress load was higher for the U21 players. In summary and based on the present observation, dynamic stress load, explosive distance, HML distance, speed intensity, total distance and total loading differentiated the external match loads between the younger age groups (U18 and U21) and first team players, being higher for the latter.

### Age-related match loads

The observation of higher match loads in the first team compared to those in younger age groups is consistent with the literature (Reynolds et al., 2021; Harkness-Armstrong et al., 2022). In contrast, although previous findings indicate external match load increases with maturity in youth players (Palucci Vieira et al., 2019; Reynolds et al., 2021; Harkness-Armstrong et al., 2022), the present observation identified many of the U21 external match load measures not to be higher, but even lower than those of the U18. To some extent, this observation may be explained by the fact that the individual match observations in the U18 compared to the U21 originate to a greater extent from offensive positions. Offensive positions are associated with higher external match loads, especially distance-related measures (Altmann et al., 2021; Forcher et al., 2022a; Rico-Gonzalez, Oliveira, Palucci; Vieria, Pino-Ortega, & Clemente, 2022). In their systematic review Palucci Vieira et al. (2019) reported increasing match running loads with aging in youth players when determined with fixed speed thresholds. On the contrary, when age-specific or individualized speed thresholds were applied, the authors identified a tendency for higher loads for younger than older players. This observation was suggested to be due to a lower technical-tactical game understanding among younger players, which may also be the case in the present observation.

The observed effects of the team (i.e., as an indicator of age group) regarding total distance (*d*: 0.49–0.94) exceeded the reported effect of tactical formation (*d*: 0.01 to 0.44; Forcher et al., 2022b). When considering the U21 and absolute values, high-speed running (*d* = 0.49 for U21 vs U18, and *d* = 0.6 for U21 vs first team, Table 8) as well as sprint distance (*r* = 0.11 and 0.2, respectively, Table 9) were affected by team assignment to a similar extent as can be the case when a pronounced effect of tactical formation was to be reported (*d* high-speed running: 0.00 to 0.73; sprint: 0.00 to 0.33; Forcher et al., 2022b). The observed effects of the team assignment on relative high-speed running and relative sprint distance, on the other hand, proved to be larger (*d* high-speed running: 0.82 and 0.89, respectively, Table 8; *r* sprint: 0.3 and 0.33, respectively, Table 9). Although the results are inconclusive, it should be considered that the degree to which the tactical formation influences the physical match load might depend on the position of the players (Forcher et al., 2022a; Forcher et al., 2022b). Effects of player position on physical match load were documented several times (Palucci Vieira et al., 2019; Altmann et al., 2021; Forcher et al., 2022a; Forcher et al., 2022b; Harkness-Armstrong et al., 2022). Thereby the documented effects differ in size, but it is known, that playing positions can explain more variance in external match loads than observed by team in this investigation (Altmann et al., 2021; Modric et al., 2022). Nevertheless, it is important to bear in mind the evidence demonstrating that the adaptation of position specific physical match performance can depend to a large extent on the individual player (Altmann et al., 2021).

### Level-dependent match loads

In case our present U21 team is interpreted as a team from a non-top league (in this case fourth highest in Switzerland), the present results fit better into the current state of literature in contrast to the age-related perspective, since some studies differentiating the physical match loads between leagues within a country, found higher loads in higher leagues. For example, Link & Anzer (2021) reported higher values of total distance (*p* < 0.001, *d* = 0.58) and running distance covered with speed >5.0 m/s (*p* < 0.001, *d* = 0.56) in the German Bundesliga compared to second German Bundesliga. Similarly, in an earlier study (Link & de Lorenzo, 2016), total distance (*p* < 0.001, *d* = 0.9) as well as the actions described as fast runs (*p* < 0.001, *d* = 1.4) and sprints (*p* < 0.001, *d* = 1.4) measured on team level were found to be higher in the German Bundesliga than in the second German Bundesliga. Greater high-intensity actions were reported from Norwegian soccer for better competitive standards than for lower leagues, while the total distance did not differ (Saeterbakken et al., 2019). Thereby some contextual factors were very similar to those of the present study. The players studied in Norway had a professional status in the first team (levels 1 and 2), whereas the team of level 4 consisted of amateurs, partly aiming for promotion and first team players returning from injuries or needing match training. Based on their findings, Saeterbakken et al. (2019) concluded that only the physical load in level 4 matches may be insufficient compared to the match loads in Level 1, which is important for coaches to recognize. Higher distance-related match loads for players who are considered to have a higher performance level compared to lower ones was also documented earlier as well as for female athletes (Mohr et al., 2003; Harkness-Armstrong et al., 2022). However, not all previous studies, e.g., some older studies from England, support these findings (Bradley et al., 2013; Di Salvo, Pigozzi, Gonzalez-Haro, Laughlin, & De Witt, 2013).

### Absolute match loads in international context

For several reasons caution is warranted when classifying the present match loads in relation to others reported in the literature. Load measures are collected with different methods (Bourdon et al., 2017; Palucci Vieira et al., 2019; Miguel et al., 2021). Some measures are based on algorithms, which may differ between manufacturers and/or depending on the user defined settings. No consensus exists regarding the definition of speed thresholds/zones, resulting in different zones being reported (Palucci Vieira et al., 2019; Miguel et al., 2021).

The present study analyzed all the measures reported by Reynolds et al. (2021) using the same GNSS technology. It is noteworthy, that the present study examined the U21 age group whereas Reynolds et al. (2021) examined U23 players. Furthermore, the present first team competed in the highest league, whereas the first team of Reynolds et al. (2021) did so in the third highest English league. Total distance of the first team, high-speed running of the U18 and the first team all appear to be higher in the present observation compared to the same teams assessed by Reynolds et al. (2021). On the other hand, explosive distance of the youth teams, high-speed running of the U23, dynamic stress load for the first team as well as HIB total distance for all teams seem higher in the English populations studied by Reynolds et al. (2022). Considering that the definition of HIB total distance from Reynolds et al. (2021) included accelerations and decelerations above 4 m/s^2^, while this study worked with 3.5 m/s^2^, these differences are particularly noticeable. Combined with the explosive distance, this may indicate different game characteristics in the two countries. The mean total distance of the present first team seems to be lower compared to German first Bundesliga (10.82 to 11.09 km; Forcher et al., 2022a) or Norwegian Soccer Level 1, 2 and 4 (11.04 to 11.15 km; Saeterbakken et al., 2019). Compared to position-specific values reported from other leagues, the mean values of distance-based measures of our first team corresponded most closely to those of player positions showing the lowest values of the respective teams. For example, in comparison to the German Bundesliga (Konefal et al., 2020; Chmura et al., 2021; Forcher et al., 2022a), the Norwegian Level 1, 2 and 4 (Saeterbakken et al., 2019) or the group stage of the UEFA Champions League (Modric et al., 2022), the mean total distance of our first team corresponds most closely to that of defenders or forwards. Comparing high-speed distance (high intensity running by Modric et al. (2022)) and sprint distance of our first team with the UEFA Champions League group stage, the values correspond most closely to those of players with central playing positions. In comparison to Norwegian soccer, the sprint distance of the first team under investigation corresponds most with those of central defenders or central midfielders at Level 1. Total distance as well as high-speed running observed for the first team in this study seem to be similar to what is known from the Croatian First Division 2019/20 season (Sekulic et al., 2021).

### Strengths, limitations and future directions

This study is the first investigating external match loads of players competing in different Swiss male soccer leagues. However, caution must be taken when generalizing the current findings, since data only from three teams of one club were available. Thus, it remains unclear whether the results of this study reflect general data for the three Swiss male soccer leagues or rather represent temporary or club-specific data. In order to understand the characteristics of leagues more generally as well as to interpret data from individual clubs in a wider context, it would be helpful if high-quality data (physical, technical, etc.) obtained using the same methodology were made available to clubs and sports scientists. Each club would benefit from such data to understand and individualize load monitoring, aiming to improve short- and long-term performance and reduce the risk of injury.

While there is no doubt accelerations and decelerations should be considered in any professional load management and thus evaluation of match loads (Harper, Carling, & Kiely, 2019; Palucci Vieira et al., 2019; Delves, Aughey, Ball, & Duthie, 2021; Miguel et al., 2021; Silva et al., 2022), it is less clear how to quantify such loads (Harper et al., 2019; Silva et al., 2022). As it is common in various studies and recommended in the literature, we employed threshold-based counts (Harper et al., 2019; Delves et al., 2021; Miguel et al., 2021). However, regarding accelerations, Sonderegger, Tschopp & Taube (2016) showed that if the running speed immediately prior to an acceleration being initiated and the maximal acceleration capacity associated with it is not considered, a number of high-intensity accelerations could be missed. I.e., arbitrarily set thresholds lead to accelerations from low speeds being overestimated and accelerations from high speeds being underestimated.

The present observation focused on external load measures, however, to provide individual recommendations throughout a training process, internal load should not be neglected, since stimulus for training (in this sense including matches) induced physiological adaptation results from the physiological load imposed on athletes (and not necessarily or lonely the external load). Ideally, an integrated approach, rigorous and consistent, combining internal and external loads is followed, as this provides more significant information about the load experienced by athletes than interpretations based on isolated data (Bourdon et al., 2017; Impellizzeri et al., 2019; Miguel et al., 2021). In addition, several studies reported physical match loads to be affected by playing positions, tactical formation, the player themselves or different contextual factors (e.g., match location, environmental conditions, match importance, preparation, fixture congestion, season phase, match outcome, nutrition strategies, game rules) (Link & de Lorenzo, 2016; Palucci Vieira et al., 2019; Julian, Page, & Harper, 2020; Altmann et al., 2021; Chmura et al., 2021; Forcher et al., 2022a; Forcher et al., 2022b; Hulton, Malone, Clarke, & MacLaren, 2022; Sarmento et al., 2022). Therefore, future studies elucidating such factors as well as integrating other components of match performance (especially technical and tactical) are warranted, to gain a holistic understanding of match performance.

## Conclusion

Based on the present data we conclude that players of different age and performance levels (U18 in highest league of their age group vs U21 in fourth highest Swiss league vs first team in highest Swiss league), show large inter-individual variability in common soccer-specific external load measures. Based on group analysis we conclude that dynamic stress load, explosive distance, HML distance, speed intensity, total distance and total loading differentiated the external match loads between the younger age groups (U18 and U21) and first team players, being higher for the latter.

The present data may support the concept to routinely monitor match loads of different age groups and competitive settings to 1) provide an indication of what players need to be prepared for, 2) track the athletic and match evolution, and 3) individually tailor training programs allowing players to fulfill the short- and long-term sport-specific requirements.

## Data Availability

The original contributions presented in the study are included in the article/supplementary material, further inquiries can be directed to the corresponding author.
